# Milk Stability: Physicochemical and Performance Parameters in Highly Technified Holstein and Jersey Herds

**DOI:** 10.1002/fsn3.71095

**Published:** 2025-10-21

**Authors:** Thais Fatima Ferreira Neves, Vivian Fischer, Fernando Batista Solano, João Ricardo Alves Pereira, Adriana de Souza Martins

**Affiliations:** ^1^ Postgraduate Program in Animal Science Ponta Grossa State University Ponta Grossa Brazil; ^2^ Departmente of Animal Science Ponta Grossa State University Ponta Grossa Brazil; ^3^ Department of Animal Science Rio Grande Do Sul Federal University Porto Alegre Brazil; ^4^ Frisía Cooperativa Agroindustrial Ponta Grossa Brazil

**Keywords:** calcium, composition, ethanol, instability, quality

## Abstract

Low stability of milk causes losses to the farmers and dairy industry by increasing milk rejection and compromising heat treatment, respectively. The stability of milk in ethanol and its relationship with the acidity and physicochemical traits of milk from Holstein and Jersey breeds were evaluated. Titratable acidity, ethanol test, ionized calcium concentration (iCa), milk chemical composition, and the milk production, parity, and body condition score of cows were analyzed. Milk characteristics and performance parameters showed that herds were of high genetic value and were raised in high‐tech systems. Milk stability was high in the two breeds (> 80% *v/v*). Milk production and composition were similar in low, medium, and high stability milk in both breeds. Augments in parity, days in milk, iCa, and acidity in the Holstein breed, and total solids in Jersey increased the occurrence of less stable milk. Ionized calcium was the main factor associated with low milk stability in both breeds.

## Graphical abstract

Unstable milk causes several problems in the dairy chain, increasing milk rejection in industrial plants. Holstein and Jersey herds, when kept under adequate nutritional, sanitary, and thermal comfort conditions, produce highly stable milk. Furthermore, the ionized calcium present in the milk is a variable strongly associated with milk instability in both breeds. Acidity was associated with milk instability in the Holstein herd, but in the Jersey herd, this association was not observed due to the higher natural acidity of milk.

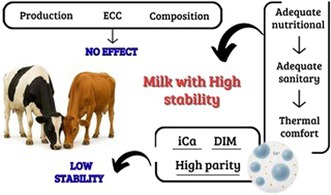

## 1 Introduction

Milk stability refers to the relative resistance of milk to withstand industrial heat treatment without coagulating. The stability of milk proteins is important for the dairy industry, especially for the manufacture of products that undergo more severe heat treatment, such as UHT milk and powdered milk (Martins et al. 2015). The use of low‐stability milk by industry may reduce yields during the processing of dairy products, as this milk may contain lower levels of lactose and protein compared with high milk stability (Oliveira et al. 2011). Furthermore, low stability can cause precipitation of milk during processing, which adheres to the equipment, thus increasing cleaning costs and milk rejection (Rosa et al. 2017). Sedimentation inside the packaging may also occur, reducing the quality of the final product.

The ethanol test has been used over the years in several countries as a parameter to determine the stability of raw milk that will be submitted to heat treatment. Ethanol acts as a dehydrating agent that causes dielectric reduction in the medium, thus reducing the negative charge of casein micelles (Nelson and Cox 2014). Milk stability is a phenomenon of multiple causes that alters the physicochemical characteristics of milk, rendering it unstable to alcohol, with acidity between 0.14 and 0.18 g/lactic acid/L; it is therefore considered nonacidic milk (Fischer et al. 2012). Unstable milk may be rejected or undervalued by the dairy industry.

In the south of Brazil, the Carambeí city and surrounding region are characterized by intensive and highly technological dairy production systems consisting of genetically selected herds, excellent nutritional and health management, and adequate thermal comfort. Some studies have evaluated the variation in the stability of raw milk (Fagnani et al. 2014; Fagnani et al. 2016) and the limitations of its use by the industry.

Different factors can alter the stability of milk, including genetics (Davis et al. 2001), breed number of lactations, lactation stage (Omoarukhe et al. 2010), nutritional factors (Gabbi et al. 2015, 2018; Garcia et al. 2023), environmental factors such as heat stress, season (Abreu et al. 2020; Garcia et al. 2024), metabolic disorders (Fagnani et al. 2014; Marques et al. 2010), and the concentration of salts in milk, particularly ionized calcium (Tsioulpas et al. 2007). There are other factors that also impact stability, such as milk pH, titratable acidity, somatic cell count, enzymatic activity, microbial load, milk protein composition, fat globule size, milking hygiene, cooling rate, storage conditions, and specific genetic variants affecting milk proteins (Brasil et al. 2015; Zanela and Ribeiro 2017; Loveday et al. 2021; Martins et al. 2024; Schmitz et al. 2024).

This study aimed to evaluate the ethanol stability of milk and its relationship with physicochemical characteristics of milk and performance parameters in Holstein and Jersey herds.

## 2 Material and Methods

The experiment was carried out on two dairy farms in the city of Carambeí, Paraná, Brazil (24°57′09.9″S and 50°07′04.6″W), from 21 January to 25 March 2020, a period comprising the summer season (southern hemisphere). The climate is classified as type Cfb during the experimental period; the average minimum temperature was 17°C and the maximum temperature was 25°C, with average precipitation of 153 mm. The farms were chosen based on the high productivity of Holstein and Jersey cattle reared in a feedlot system, being representative of the dairy farms in the region.

### 2.1 Description of the Holstein Herd—Tie‐Stall System

Sixty‐three high‐producing Holstein cows (mean production of 39.3 ± 10.4 L/cow/day) kept in a tie‐stall feedlot system were evaluated. The cows were housed in two completely closed barns equipped with a forced ventilation system (evaporative panels, fans, and exhaust fans). The mean temperature in the barn was 21.6°C, and the relative humidity was 81%, both monitored with an electronic panel. Rubber mattresses were used as bedding material, and lime was used for cleaning.

Milk samples were collected from each cow every 2 weeks at four time points, totaling 252 samples; 42% of the samples were from primiparous cows and 58% from multiparous cows (second to seventh lactation). The mean number of days in milk (DIM) of the herd was 175.1 ± 155.

The animals were reared in a tie stall system and milked three times a day at 5:00, 14:00, and 22:00 h. For milk sample collection, four graduated gauges were attached to the milking units, and samples were collected at the end of milking. At the end of milking, the graduated gauges were relocated to the next milking units so that the milk from all the cows was sampled. The diet was formulated to meet the nutritional requirements of cows with a mean production of 40 L/cow/day (NRC 2001). Water was available *ad libitum*.

### 2.2 Description of the Jersey Herd—Compost Barn System

Seventy‐one lactating cows of the high‐producing Jersey breed (mean production of 25 ± 8.3 L/cow/day) were evaluated. The animals were kept in a compost barn system, with an area of 14 m^2^ per animal. Sawdust was used as bedding for composting waste, turned twice a day. The barn was divided into an area destined for feeding and another area for collective rest, where the animals remained between milkings. The facility was equipped with a low‐speed ceiling ventilation system.

The cows were milked in a milking parlor with a pit, with eight milking units. To collect milk, eight milk sampling cups were attached to each milking unit. Three fortnightly samplings were carried out, totaling 213 samples; 47.4% of the samples were from primiparous cows and 52.6% from multiparous cows (two to five lactations), with a mean number of DIM of 131.9 ± 73.6. The cows were milked twice a day at 4:00 and 14:30 h. The diet was formulated to meet the nutritional requirements of cows with a mean production between 18 and 28 L/cow/day (NRC, 2001). Water was provided *ad libitum*.

### 2.3 Milk Samples

Samples were collected every 2 weeks at the afternoon milking of the two herds into identified 200‐mL plastic bottles without preservatives using automated collectors. The samples were stored in thermal boxes with ice and transported to the laboratory of the State University of Ponta Grossa for analysis of stability, titratable acidity, pH, and Ca^2+^. The milk samples were stored under refrigeration at 4°C for approximately 11 h and then opened to permit evaporation of carbon dioxide.

### 2.4 Laboratory Analyses

#### 2.4.1 Titratable Acidity

Titratable acidity (g lactic acid/L) was determined using the method described by Tronco (2010). The samples were classified according to acidity as follows: a) milk with normal acidity: 0.14–0.18 g lactic acid/L; b) acid milk: acidity > 0.18 g lactic acid/L; c) alkaline milk: acidity < 0.14 g lactic acid/L.

#### 2.4.2 Ethanol Test

The milk stability test was performed as described by Tronco (2010) using the following ethanol concentrations (*v/v*): 72%, 76%, 78%, and 80%. Milk stability was assessed based on visual observation of the presence of clots or lumps. Milk stability was registered as the lowest ethanol concentration that induced clotting. Samples that did not clot using the 80% *v/v* ethanol solution were considered to be stable at 82% *v/v*. Based on these values, the milk samples were classified into three stability levels: high: samples with ethanol stability > 80% *v/v*; intermediate: samples with ethanol stability of 78%–80% *v/v*; low: samples with ethanol stability of 72%–76% *v/v*.

#### 2.4.3 Ionized Calcium Concentration

Ionized calcium (iCa) concentrations in milk were analyzed using an Orion digital potentiometer with ion‐selective electrodes (Barros et al. 1999). After collection, the samples were kept refrigerated for 11 h at an average temperature of 4°C. Immediately after sample preparation, the electrode was inserted to quantify iCa (mg/L) in a mixture of milk and ISA solution (50:1).

#### 2.4.4 Cryoscopy

The freezing point (FP) of milk was analyzed with a Minilak cryoscope according to the manufacturer's instructions. The results were expressed as degrees Hortvet (^o^H).

#### 2.4.5 Chemical Analysis of Milk

Chemical analyses were performed monthly by the Paraná Association of Holstein Breeders (APCBRH). Protein, fat, lactose, and total solids (TS) were analyzed with an infrared spectrophotometer (B 2300 Combi, Bentley Instruments) (AOAC 2016). The somatic cell count (SCC) was determined by flow cytometry (Somacount‐500, Bentley Instruments 1994).

### 2.5 Evaluation of Body Condition Score

The body condition score (BCS) was evaluated every 2 weeks on both farms before the afternoon milking using the method proposed by Edmonson et al. (1989). A scale ranging from 1 – 5, with 0.25‐point increments, was used. Two trained evaluators performed the assessments.

### 2.6 Evaluation of Somatic Cell Score

The SCC was transformed into a linear somatic cell score (SCS) according to the method of Dabdoub and Shook (1984).

### 2.7 Statistical Analysis

It was evaluated 465 samples of milk, 213 of which were from the Jersey herd and 252 from Holstein herds. The correlations between variables were analyzed using the PROC CORR procedure (Spearman's coefficient) of SAS. Data were classified by breed, and statistical analyses were performed separately within each breed, without comparing them.

The linear association of milk stability with the independent variables [parity number (Nparity), DIM, TS concentration, SCC, BCS, ethanol test stability (ranging from 72% to 82%), ionized calcium (iCa) concentration, titratable acidity, and cryoscopy] was tested using the REG procedure of SAS (stepwise selection option).

Multivariate analysis of principal factors (PFs) was used to analyze the association of variables in the dispersion of data. First, the data were standardized using the STANDARD procedure, and the values were transformed considering a mean = 0 and a standard deviation = 1, thus reducing the effect of unequal magnitudes between the original variables. The Measurement System Analysis (MSA) option was used to select the variables that would be kept in the model for PF analysis. The following variables were retained: Nparity, DIM, TS concentration, SCC, BCS, milk stability at the cited concentrations, iCa concentration, titratable acidity, and cryoscopy (MSA > 0.4). The PFs were considered significant when they exhibited eigenvalues ≥ 1.0. Individual values (of each cow × day combination) of the PF scores were calculated using the SCORE procedure of SAS.

Logistic regression was performed to determine the original variables and PFs that increased the risk of occurrence of low stability using the LOGISTIC procedure of SAS (backward option).

The milk composition, body condition, and reproductive traits of milk‐producing cows of different stability classes were submitted to analysis of variance using the GLM procedure of SAS and the LSmeans option for the separation of means. The results of SCC analysis were log10‐transformed for statistical analysis. A *p* value < 0.05 was considered significant.

## 3 Results

### 3.1 Holstein Breed

Approximately 72.6% of the milk samples did not clot in the 80% (*v/v*) ethanol test and were classified as highly stable. Only 13.9% and 13.5% of the milk samples showed intermediate and low stability, respectively. There was no difference (*p* > 0.05) in milk production, BCS, fat, protein, TS, or acidity between stability levels (Table 1).

**TABLE 1:** Mean values of number parity (Nparity), milk production (MP), days in milk (DIM), body condition (BCS), fat, protein, lactose, total solids (TS), somatic cells score (SCS), milk stability in ethanol (STB), ionized calcium (iCa), titratable acidity (TA), and freezing point (FP) in milk samples of Holstein cows, depending on the level of stability (High, intermediate, and low).

Mean				Stability			*P*	Standard deviation
	*n*	High	*n*	Intermediate	*n*	Low		
Nparity	83	2.36b	35	2.54ab	34	3.35a	0,0126	1.79
MP (L/cow/day)	183	40.1	35	39.8	34	35.7	NS	10.55
DIM (days)	183	186.3b	35	219.1ab	34	278.7a	0.0035	1.41
BCS	183	3.17	35	3.19	34	3.06	NS	0.64
Fat (g/100 g)	183	3.53	35	3.83	34	3.74	NS	0.84
Protein (g/100 g)	183	3.36	35	3.35	34	3.48	NS	0.32
Lactose (g/100 g)	183	4.76a	35	4.71a	34	4.49b	< 0.001	0.23
TS (g/100 g)	183	12.5	35	12.7	34	12.6	NS	1.05
SCS (cell/log^10^)	183	1.51a	35	1.73b	34	1.73b	0.0090	0.37
STB (% *v/v*)	183	82a	35	79.2b	34	73.1c	< 0.001	3.11
iCa (mg/L)	183	80,8a	35	91.6b	34	104.0c	< 0.001	25.13
TA (g lactic acid/100 mL milk)	183	15.3	35	15.3	34	15.6	NS	0.15
FP (°H)	183	−0.543	35	−0.544	34	−0.54	NS	0.008

*Note:* Different letters on the same line: significant difference between levels. P: significant level. High stability: above 82% *v/v*; Intermediate stability: between 78%–80% *v/v*; Low stability: between 72%–76% *v/v*.

Abbreviation: *N*, Number of observations.

Milk samples with high stability originated from animals with fewer calvings and fewer DIM compared to samples with intermediate and low stability. High‐ and intermediate‐stability samples exhibited a higher concentration of lactose compared to low‐stability samples (*p* < 0.05). High‐stability milk samples had lower SCS and iCa concentrations than intermediate‐ and low‐stability samples.

Stability was negatively correlated (*p* < 0.05) with Nparity, DIM, and Ca^2+^ (Table 2). Simple linear regression between stability level (Y) and the original variables revealed that stability was reduced by DIM and iCa and increased by lactose content according to the following equation: Y = 60.9–0.003 DIM + 4.9 lactose—0.04 iCa (*p* < 0.01, R^2^ = 0.28).

**TABLE 2:** Correlation levels between the number of parity (Nparity), days in milk (DIM), total solids (TS), somatic cell count (SCC), milk stability in ethanol (STB), ionized calcium (iCa), titratable acidity (TA), and freezing point (FP) in milk samples of Holstein cows.

	Nparity	DIM	TS	SCC	BCS	STB	iCa	TA	FP
Nparity DIM ST SCC BCS STB iCa FP	1	0.10 1	0.01 −0.04 1	**0.25** * 0.17 0.07 1	0.05 −0.07 0.12 0.02 1	**−0.22** * **−0.21** * 0.04 −**0.17** * **0.12** * 1	−0.12 −0.06 0.09 0.03 0.02 **−0.32** * 1	**−0.33** * 0.01 0.44 −**0.11** **0.26** * −0.18 −0.08 1	−0.01 **−0.23** * −0.19 −0.02 0.01 −0.02 −0.12 0.02 1

* Significant correlations. NPARITY: number of parity.

Three PFs were significant (eigenvalues ≥ 1.0) and explained 49.7% of the total variance (Table 3). In PF1, the original variables with the highest factor loadings were TS, BCS, and acidity, which showed a positive association with each other. In PF2, the original variables with the highest factor loadings were Nparity, DIM, and SCC, which were positively associated with each other. In PF3, the original variables with the highest factor loadings were milk stability and iCa, showing a strong negative association with each other.

**TABLE 3:** Factor load, communality, and percentage of variance of the variables used for the factor analysis that relates to the occurrence of milk stability of Holstein cows.

Parameter	Principal factor			Communality
	Factor 1	Factor 2	Factor 3	
Nparity	−0.30481	**0.69828** *	−0.03179	0.58
DIM (days)	−0.03352	**0.63740** *	0.24952	0.46
BCS	**0.65815** *	−0.00318	−0.23699	0.48
TS (g/100 g)	**0.79610** *	0.03550	0.14097	0.65
SCC (x mil cell/mL)	0.06667	**0.73534** *	−0.02058	0.54
STB (% *v/v*)	0.07699	−0.27614	**−0.70623** *	0.58
iCa (mg/L)	0.04756	−0.10306	**0.80467** *	0.66
TA (g lactic acid/100 mL milk)	**0.79227** *	−0.19409	0.10108	0.67
FP (°H)	−0.27970	−0.34468	−0.33324	0.30
Variance %	18.79	16.79	14.07	

Abbreviations: BCS: body condition score; DIM: days in milk; EST: milk stability to ethanol; FP: freezing point; iCa: ionized calcium concentration; SCC: somatic cell count; TA: titratable acidity; Nparity: number of parity; TS: total solids.

* Variables responsible for most of the variance retained in factor analysis.

PF2 and PF3 were retained in the logistic regression model of individual scores (observations) to assess the risk of a sample exhibiting low stability (Table 4). PF3 showed a greater association with the risk of occurrence of low stability. The original variables Nparity, DIM, iCa, and acidity were retained in the logistic regression model and were positively associated with the occurrence of low milk stability. The original variable showing the greatest association was titratable acidity.

**TABLE 4:** Logistic regression analysis relating the variables retained in the factor analysis and original variables in the odds of occurrence of low milk stability (≤ 76% *v/v*) in Holstein cows.

Variable	Odds ratio	CI 95%	*P*
PF2	2.508	1.306–4.816	0.0057
PF3	18.516	6.996–49.217	< 0.0001
Nparity	1.469	1.161–1.859	0.0014
DIM	1.004	1.001–1.007	0.0025
iCa	1.033	1.017–1.05	< 0.0001
Titratable acidity	19.92	1.414–280.87	0.0267

*Note:* CI, confidence interval; DIM, Days in milk; iCa, ionic calcium concentration; Nparity, number of parity; PC, principal component.

### 3.2 Jersey Breed

Approximately 57%, 29%, and 13% of the samples were classified as high, intermediate, and low stability, respectively. There was no difference in Nparity, milk production, DIM, BCS, protein, lactose, SCS, or cryoscopy between stability levels (Table 5). iCa concentrations were lower in high‐stability milk compared to intermediate‐ and low‐stability samples. Furthermore, high‐stability samples exhibited lower concentrations of fat and TS than intermediate‐ and low‐stability samples.

**TABLE 5:** Mean values of number parity (Nparity), milk production (MP), days in milk (DIM), body condition (BCS), fat, protein, lactose, total solids (TS), somatic cells score (SCS), milk stability in ethanol (STB), ionized calcium (iCa), titratable acidity (TA), and freezing point (FP) in milk samples of Jersey cows, depending on the level of stability (high, intermediate, and low).

Mean			Stability				*P*	Standard deviation
	*n*	High	*n*	Intermediate	*n*	Low		
Nparity	122	2.09	62	2.25	29	2.27	NS	1.39
MP (L/cow/day)	122	25.7	62	26.2	29	25.6	NS	8.63
DIM (days in milk)	122	161.9	62	167.9	29	173.6	NS	70.5
BCS	122	3.25	62	3.22	29	3.18	NS	0.52
Fat (g/100 g)	122	4.50b	62	4.88ab	29	5.14a	0.038	1.38
Protein (g/100 g)	122	3.82	62	3.91	29	3.92	NS	0.41
Lactose (g/100 g)	122	4.70	62	4.66	29	4.63	NS	0.26
TS (g/100 g)	122	13.9b	62	14.3ab	29	14.6a	0.037	1.49
SCS (log10)	122	1.81	62	1.81	29	1.71	NS	0.34
STB (% *v/v*)	122	82a	62	78.9a	29	73.7b	< 0.001	2.95
iCa (mg/L)	122	81.5b	62	93.7a	29	95.0a	< 0.001	19.41
TA (g lactic acid/100 mL milk)	122	15.9a	62	15.9a	29	14.4b	0.007	0.19
FP (°H)	122	−0.543	62	−0.543	29	−0.543	NS	0.008

*Note:* Different letters on the same line: significant difference between levels. *P*: significant level. High stability: above 82% *v/v*; Intermediate stability: between 78%–80% *v/v*; Low stability: between 72%–76% *v/v*.

Stability was negatively correlated (*P* < 0.05) with TS and iCa and positively correlated (Table 6).

**TABLE 6:** Correlation levels between the number of parity (Nparity), in milk samples of Jersey cows.

	NPARITY	DIM	TS	SCC	BCS	STB	iCa	TA	FP
NPARITY	100	0.22	−0.19	0.10	−0.17	0.01	0.11	**−0.33** *	0.11
DIM		100	**0.27** *	0.04	0.03	−0.11	−0.07	0.16	0.01
TS			100	0.20	0.05	−**0.20** *	−0.06	0.04	−0.01
SCC				100	−0.02	0.14	−0.01	−0.08	−0.10
BCS					100	0.01	**−0.17** *	−0.07	−0.05
STB						100	**−0.26** *	**0.20** *	0.02
iCa							100	−0.01	−0.10
TA								100	−0.09
FP									100

Abbreviations: DIM, days in milk; FP, freezing point; Ica, ionized calcium; SCC, somatic cell count; STB, milk stability in ethanol; TA, titratable acidity; TS, total solids.

* Significant correlations. NPARITY: number of parity.

Five PFs were significant and explained 64.8% of the total variance (Table 7). In PF1, the original variables with the highest factor loadings were Nparity, acidity, and SCC, with a negative association between Nparity and acidity. In PF2, the variables with the highest factor loadings were DIM and TS, with a positive association between these variables. In PF3, the variables with the highest factor loadings were milk stability and iCa, with a strong negative association for iCa. In PF4, the variables with the highest factor loadings were SCS and iCa, showing a negative association. In PF5, the variables with the highest factor loadings were SCC and cryoscopy, with SCS showing a negative association.

**TABLE 7:** Factor load, communality, and percentage of variance of the variables used for the factor analysis that relates to the occurrence of milk stability of Jersey cows.

Parameter	Principal factor					communality
	Factor 1	Factor 2	Factor 3	Factor 4	Factor 5	
NPARITY	**−0.71071** *	0.04009	−0.01028	−0.38930	0.13054	0.67
DIM (days)	−0.00670	**0.80557** *	−0.00401	−0.13387	0.11148	0.67
BCS	0.03069	0.01942	0.05702	**0.86379** *	−0.02831	0.75
TS (g/100 g)	0.12139	**0.73655** *	−0.13102	0.23318	−0.16770	0.65
SCC (x mil célls/mL)	**−0.41290** *	0.32388	0.36343	−0.04410	**−0.61604** *	0.78
STB (% *v/v*)	0.13476	−0.25787	**0.81795** *	−0.08098	−0.05406	0.76
iCa (mg/L)	−0.09890	−0.15587	**−0.64312** *	**−0.41349** *	−0.19811	0.65
TA (g lactic acid/100 mL milk)	**0.80766** *	0.17901	0.24329	−0.18996	−0.04662	0.78
FP (°H)	−0.26380	0.08193	0.16407	−0.04309	**0.79132** *	0.73
%Variance	14.41	14.27	13.21	11.87	11.08	

Abbreviations: BCS, body condition score; DIM, days in milk; FP, freezing pointHigh stability, above 82% *v/v*; iCa, ionized calcium concentration; SCC, somatic cell count; STB, milk stability to ethanol; Nparity, number of parity; TS, total solids.

* Variables responsible for most of the variance retained in factor analysis. Titratable acidity. Intermediate stability: between 78%–80% *v/v*; Low stability: between 72%–76% *v/v*; P: significant level.

PF3, PF4, and PF5 were retained in the logistic regression model (Table 8).

**TABLE 8:** Logistic regression analysis relating the variables retained in the factor analysis and original variables in the occurrence of low milk stability in Jersey cows.

Parameter	Odds ratio	*CI 95%*	*p*
PF 3	0.007	0.001–0.058	< 0.0001
PF 4	3.73	1.219–11.455	0.0211
PF 5	5.118	1.218–21.503	0.0211
TS (g/100 g)	1.654	1.194–2.0291	0.0025
SCS (log 10)	0.192	0.056–0.660	0.0088
TA (g lactic acid/100 mL milk)	0.004	0.001–0.053	< 0.0001

Abbreviations: CI: confidence interval; PF: principal factor; SCS: Somatic cell score; TA: Titratable acidityHigh stability: above 82% *v/v*; TS: total solids.

PF3 reduced the risk of milk exhibiting low stability, while PF4 and PF5 increased the risk of low stability. Among the original variables retained in the regression analysis, TS concentration increased the risk of the occurrence of low milk stability.

## 4 Discussion

The Holstein and Jersey cows evaluated in the present study were adequately managed concerning nutrition, thermal comfort, and welfare conditions, thus achieving high productivity and milk quality. Approximately 90% of the milk samples had a SCC less than 200 × 10^3^ cells/mL (Tables 1 and 5), indicating adequate udder health.

Milk stability was high, as more than 71% and 81% of milk samples presented stability higher than 80% *v/v* in Jersey and Holstein, respectively; agreeing with previous studies that evidenced the positive effect of nutrition (Gabbi et al. 2018), animal health (Fagnani et al. 2014), and thermal comfort (Abreu et al. 2020) on milk stability. Voges et al. (2018) observed that milk with low stability (72% *v/v*) is more frequent on smallholder farms that use less technology.

Research evaluating aspects related to the production and composition of milk from Holstein and Jersey cows, such as number and stage of lactation, parity, and climate conditions, has shown that variations occur due to physiological processes inherent to each breed (Bangani et al. 2022). Few studies have investigated breed differences regarding milk stability, for example, those comparing Holstein, Gyr, and Girolando animals (Botaro et al. 2007; Vizzotto et al. 2021). Lim et al. (2020), analyzing the milk composition from Holstein and Jersey cows at the beginning of lactation, found that the fat, protein, and citrate contents were higher in Jersey milk. Regarding milk coagulation potential, this was higher in Jersey milk than in Holstein milk, suggesting that Jersey cow milk is more effective in dairy product processing than Holstein cow milk under the same nutritional and environmental conditions. Schmitz et al. (2024) evidenced that characteristics related to the milk's ability to be processed at the dairy industry, such as thermal stability, ionic calcium, and acidity, varied between genetic groups (Holstein, Jersey, and their crossbreds) and lactation stages. Therefore, understanding which factors affect milk processing can be useful for better selecting milk on the farm, remembering that milk properties will not always predict how it will behave during processing.

In the present study, Holstein cows producing milk with the highest stability showed shorter DIM, lower parity, acidity, and iCa concentration, while Jersey cows producing milk with the highest stability also presented the lowest fat, total solids, and iCa concentrations. Thus, iCa concentration was the only variable shared by the two breeds and affected linearly and negatively stability, confirming previous studies about the negative effects of iCa concentration on milk stability (Tsioulpas et al. 2007). Ionized calcium decreases the ability of caseins to maintain their structure, reducing electrostatic repulsion and causing the aggregation of micelles (Philippe et al. 2003) and consequent milk clotting.

The results of logistic regression of factor loadings of original variables (Tables 4 and 8) confirm these results, for example, the association between PF3 with the occurrence of low stability, evidencing the negative association between stability and iCa. According to Faria et al. (2017), electrical conductivity is lower in unstable milk, possibly due to the higher concentration of iCa in milk. Furthermore, a positive association between acidity and the occurrence of low stability was observed only in the Holstein breed. At acid pH (< 6.5), changes occur in the balance of ions in order to maintain the micellar structure, with a consequent increase in the passage of calcium from the colloidal to the soluble phase, increasing the concentration of iCa in milk and causing the destabilization of micelles and their precipitation (Singh 2004).

In the Jersey breed, high‐stability samples were associated with low Ca^2+^ concentration, lower TS content, and higher acidity, but not with lactation stage (DIM) or parity (Tables 5 and 6). The high TS content of milk from Jersey cows may increase the natural acidity of milk without altering pH (Durr et al. 2001). Similar results have been reported by Oliveira et al. (2011), who found lower fat and TS contents in ethanol‐stable samples. In Jersey cows, acidity was lower in low‐stability milk, probably related to the lower protein and phosphate concentrations, although we acknowledge we have no measured casein, phosphates, and citrate, and we recognize this as a limitation.

The negative relationship of advanced lactation stage and SCS with stability observed with Holstein in the present study might be explained by the fact that these factors can modify the concentration of ions in milk, increasing iCa. As lactation progresses, epithelial desquamation and tight junction permeability increase in the mammary gland (Barros 1999). Reduced milk secretion and the increased cellular transport of components from blood to milk at the end of lactation alter salt concentrations and can reduce stability (Singh 2004). Marques et al. (2010) evaluated the effect of two diets (high and low supplementation with concentrate) on cows in an advanced stage of lactation and did not find any improvement in milk stability. The authors attributed this result to changes in the salt balance of milk that occur in cows in the final stage of lactation. Thus, the greater parity and advanced lactation (longer DIM) observed in this study may have reduced milk stability, agreeing with Pereira (2019) that the frequency of unstable milk was highest in cows with more than four parturitions.

The lower lactose levels in low‐stability milk observed only in Holstein cows may be explained by the more advanced DIM and higher SCS compared to high‐stability samples (Table 1). Higher SCC may be related to the low stability of milk due to proteolysis, low casein content, and high sodium and chlorine levels. These compounds cause the destabilization of the casein micelles (Horne 2016), promoting the formation of clots. Inflammation of the mammary gland caused by mastitis increases the permeability of blood vessels, which, in turn, leads to an increase in milk SCC and serum proteins (Santos and Fonseca 2007). Furthermore, changes in permeability can cause a decrease in lactose, protein, and milk production, compromising the milk yield of the industry (Santos and Fonseca 2019).

A recurring problem with ethanol‐unstable milk is that it might be rejected by the dairy industry. This is related to the fact that, with increasing temperature during heat treatment, particularly the production of powdered and UHT milk, casein instability can cause coagulation, leading to major disruptions during processing. Although unstable milk cannot withstand intense thermal processing, it can be used in milder heat treatments such as pasteurization and in the manufacture of other products such as dairy beverages (Backes et al. 2012).

## 5 |Conclusions

Holstein and Jersey herds kept under adequate nutritional, sanitary management, and thermal comfort conditions produced milk with high stability. Cows' BCS, milk yield, and composition were not affected by stability in either breed. Variables affecting stability changed according to the breed of cows. In the Holstein breed, low stability was associated with advanced DIM, parity number, and acidity, but not in the Jersey herd. Ionized calcium was the only variable associated with stability in both breeds, demonstrating the importance of this parameter in the assessment of milk stability.

## Author Contributions

**Thais Fatima Ferreira Neves:** conceptualization, data curation, formal analysis, investigation, supervision, methodology, visualization and ilustration, project administration, writing – original draft and editing. **Vivian Fischer:** formal analysis, supervision, conceptualization, data curation, writing and review. **Fernando Batista Solano:** formal analysis, investigation, methodology and review. **João Ricardo Alves Pereira:** conceptualization, validation and review. **Adriana de Souza Martins:** supervision, validation, writing, review, funding acquisition, methodology and project administration.

## Ethics Statement

This manuscript used milk samples collected during the routine milking process of the animals, without interfering with the normal milking procedure. Therefore, the study did not need approval from the animal welfare ethics committee.

## Conflicts of Interest

The authors declare no conflicts of interest.

## Data Availability

The data that support the findings of this study are available from the corresponding author upon reasonable request.
